# Nurse–nurse collaboration among millennial emergency room nurses in government hospitals in Metro Manila, Philippines: a cross-sectional study

**DOI:** 10.1186/s12912-026-04923-2

**Published:** 2026-06-22

**Authors:** Ma. Jessica Zyra Cunanan, Diannie Buensalida, Jamaica Capunong, Anjelina Mey Gozo, Rica Ella Tolentino, Clarence Marie Nava, John Eric Rosales

**Affiliations:** 1 College of Nursing, Pamantasan ng Lungsod ng Marikina, Marikina City, Metro Manila, Philippines; 2 College of Nursing, Lyceum Technological College, Tiaong, Quezon Philippines

**Keywords:** Nurse–nurse collaboration, Millennial nurses, Emergency nursing, Teamwork, Nursing workforce, Philippines

## Abstract

**Background:**

Effective collaboration among nurses is essential for delivering safe and high-quality patient care, particularly in high-acuity environments such as emergency departments. Ineffective collaboration may lead to poor communication, mistrust among team members, and an increased risk of medical errors. Millennials currently comprise a substantial proportion of the global nursing workforce and bring unique communication styles, work values, and career expectations into clinical practice. However, limited evidence exists regarding nurse–nurse collaboration among millennial emergency room nurses in the Philippine healthcare setting.

**Methods:**

A quantitative descriptive cross-sectional study was conducted among millennial emergency room nurses working in five government hospitals in Metro Manila, Philippines. A total of 173 respondents were selected from 194 eligible nurses through simple random sampling, yielding a response rate of 89.2%. Data were collected using the Nurse–Nurse Collaboration (NNC) Scale, a validated 22-item instrument measuring four domains: conflict management, common goals, communication and coordination, and professionalism and autonomy. Descriptive statistics, weighted mean, Fisher’s exact test, and Pearson correlation were used to analyze the data.

**Results:**

The largest proportion of respondents were 30 years old and below (38.7%), female (68.8%), and had 2–9 years of clinical experience (76.9%). Respondents reported favorable perceptions of nurse–nurse collaboration across all domains including conflict management (M = 1.556), common goals (M = 1.550), communication and coordination (M = 1.633), and professionalism and autonomy (M = 1.586). No significant relationship was found between collaboration and demographic factors such as age and gender (*p* > .05). However, length of experience showed weak but statistically significant negative correlations with collaboration in terms of common goals (*r* = − .161, *p* = .034) and professionalism and autonomy (*r* = − .178, *p* = .019).

**Conclusions:**

Millennial emergency room nurses demonstrated favorable perceptions of nurse–nurse collaboration across all domains. While age and gender were not significantly associated with collaboration, clinical experience demonstrated weak negative relationships with selected domains of nurse–nurse collaboration. The findings may support efforts to strengthen collaborative workplace cultures and professional development initiatives within emergency nursing environments.

## Background

Effective collaboration among healthcare professionals is essential for ensuring safe and high-quality patient care. Poor communication and ineffective teamwork have been consistently identified as major contributors to adverse events in healthcare settings [1, 2]. In high-acuity environments such as emergency departments, where rapid decision-making and coordinated clinical actions are required, collaboration among nurses is particularly critical for maintaining patient safety and efficient care delivery [3].

Nurse–nurse collaboration refers to the cooperative interaction between nurses working together toward shared clinical goals, characterized by effective communication, mutual respect, shared decision-making, and professional autonomy [4]. Previous research has demonstrated that collaborative nursing environments are associated with improved patient outcomes, higher job satisfaction, and reduced burnout among nurses [5, 6]. Conversely, poor collaboration among nurses may lead to workplace conflict, decreased morale, and compromised quality of care [7]. Recent studies have further emphasized the importance of healthy work environments, resilience, and organizational support in promoting teamwork and professional collaboration among nurses [8–11].

Healthcare organizations today are increasingly characterized by generational diversity in the workforce. The nursing profession currently includes multiple generations, including Traditionalists, Baby Boomers, Generation X, Millennials, and Generation Z [12]. Among these groups, Millennials—individuals born between 1981 and 1996—represent a growing proportion of the global nursing workforce [13]. Millennial nurses are often described as technologically adept, collaborative in their approach to work, and motivated by opportunities for professional development and supportive work environments [14].

Despite these positive attributes, generational differences may also create challenges in workplace communication and teamwork. Studies have suggested that variations in work values, communication styles, and expectations between generations can influence collaboration within healthcare teams [15]. Understanding how millennial nurses interact and collaborate with their colleagues is therefore essential for creating supportive and effective clinical environments.

Emergency departments present unique challenges for teamwork and collaboration. Nurses working in emergency settings must manage unpredictable workloads, critically ill patients, and complex clinical situations that require rapid interdisciplinary coordination [16]. In such contexts, effective nurse–nurse collaboration plays a crucial role in ensuring timely interventions and maintaining patient safety [17].

The present study is guided by Social Learning Theory, which proposes that human behavior develops through interactions between personal experiences, environmental influences, and observed behaviors [18]. Within healthcare organizations, nurses may develop collaborative practices by observing colleagues, learning from shared experiences, and adapting to the norms of the workplace environment.

Social Learning Theory emphasizes that individuals acquire behaviors through observation, imitation, reinforcement, and interaction within social environments [18]. In nursing practice, collaborative behaviors may develop through professional role modeling, shared clinical experiences, and continuous interaction within healthcare teams. Emergency room nurses working in high-acuity environments are frequently exposed to teamwork behaviors that shape communication patterns, conflict management approaches, and professional decision-making. Through repeated exposure to collaborative workplace norms and interactions with colleagues, nurses may strengthen collaborative competencies necessary for effective patient care delivery.

Recent evidence also suggests that prosocial workplace behaviors and communication practices may strengthen teamwork and collaborative interactions among healthcare professionals, particularly in high-pressure clinical environments [19].

Although several studies have explored collaboration among nurses in various healthcare contexts, limited research has examined nurse–nurse collaboration specifically among millennial emergency room nurses in the Philippine healthcare setting. Understanding collaborative dynamics within this population may provide valuable insights for nursing leaders, healthcare administrators, and policymakers seeking to strengthen teamwork and improve patient outcomes in emergency care environments.

Therefore, this study aimed to assess nurse–nurse collaboration among millennial emergency room nurses in selected government hospitals in Metro Manila and determine whether demographic factors influence collaborative behaviors.

## Methods

### Study design

This study utilized a quantitative descriptive cross-sectional research design to assess nurse–nurse collaboration among millennial emergency room nurses.

### Study setting

The study was conducted in five government hospitals in Metro Manila. The participating hospitals were tertiary-level government institutions with estimated bed capacities ranging from approximately 200 to 500 beds and provided high-volume emergency healthcare services to diverse patient populations within Metro Manila. These hospitals provide emergency and acute healthcare services to large and diverse patient populations and manage high patient volumes requiring rapid clinical decision-making and coordinated interdisciplinary care. Government emergency departments in Metro Manila commonly encounter complex healthcare demands, making effective nurse–nurse collaboration essential for maintaining patient safety and efficient healthcare delivery.

### Participants and sampling

Participants were millennial nurses aged 28–43 years currently employed in emergency departments of government hospitals in Metro Manila and with at least six months of clinical experience.

Simple random sampling was used to select participants from the target population. Eligible participants were identified through hospital nursing rosters obtained with institutional permission. All nurses who met the inclusion criteria were assigned unique identification numbers. A sampling frame was created from the list of eligible nurses across the participating hospitals. Using a computer-generated random number selection procedure, identification numbers were randomly selected until the required sample size was achieved. This approach ensured that each eligible nurse had an equal probability of being included in the study. Recruitment was subsequently coordinated with nursing supervisors from participating hospitals. A sample size of 173 respondents was determined using G*Power statistical software with the following parameters:

- significance level (*α*) = 0.05
- effect size = 0.30 (medium)
- statistical power = 0.95

These parameters ensured adequate statistical power for detecting potential associations between demographic variables and nurse–nurse collaboration domains.

A total of 173 nurses participated in the study out of 194 eligible nurses, yielding a response rate of 89.2%.

### Instrument

Data were collected using an adapted version of the Nurse–Nurse Collaboration Scale developed by Dougherty and Larson [4]. The instrument was adapted from the original scale with appropriate acknowledgment of the source, and modifications were made to ensure contextual relevance for emergency room nurses in the Philippine setting. The original instrument was designed to measure collaboration among nurses across multiple domains, including communication, coordination, professionalism, shared processes, and conflict management. The Nurse–Nurse Collaboration Scale has been previously validated and widely used to assess collaborative behaviors among nurses [4].

The adapted questionnaire consisted of 22 items distributed across four domains. Responses were measured using a five-point Likert scale ranging from 1 (Always) to 5 (Never). The original version of the instrument contained approximately 35 items distributed across several collaboration domains. For the present study, selected items were modified or excluded to improve contextual relevance and applicability to emergency room nursing practice in the Philippine healthcare setting. Adaptations included refinement of wording and modification of response descriptors to improve clarity and contextual appropriateness for respondents. The questionnaire was translated into Filipino by a language expert to improve linguistic and contextual appropriateness for respondents. The translated version was reviewed by nursing professionals to ensure conceptual consistency with the original instrument. The adapted instrument retained the core structure of the original scale while incorporating contextual modifications relevant to the local emergency room setting.

### Validity and reliability

Content validity of the adapted instrument was evaluated by three nursing experts with experience in clinical nursing practice and nursing research. The experts reviewed the questionnaire for clarity, relevance, and contextual appropriateness within emergency nursing settings. Revisions were incorporated based on expert recommendations prior to pilot testing. A formal Content Validity Index (CVI) was not computed during the original instrument adaptation process; however, expert evaluation focused on the clarity, relevance, and contextual appropriateness of the adapted items for emergency room nursing practice. A pilot study was subsequently conducted to assess reliability and internal consistency of the adapted instrument. Cronbach’s alpha for the questionnaire was 0.749, indicating acceptable internal consistency. Domain-specific reliability coefficients were not separately computed in the original dataset and were therefore unavailable for further analysis in the present revision.

### Data collection

Data were collected between March and June 2024 using both face-to-face surveys and online questionnaires administered through Google Forms. Prior to data collection, permission was obtained from institutional authorities, and ethical clearance was secured from the Institutional Ethics Review Board of Pamantasan ng Lungsod ng Marikina. Participants were provided with written informed consent forms explaining the purpose of the study, potential benefits, voluntary nature of participation, and their right to withdraw at any time without penalty.

Face-to-face surveys were administered within hospital settings at schedules coordinated with nursing supervisors to minimize disruption of clinical duties. Online questionnaires were distributed through official communication channels accessible only to eligible participants. To minimize duplicate responses, participants were instructed to complete the survey only once and submissions were monitored during the data collection process.

### Data analysis

Data were analyzed using descriptive and inferential statistical techniques. Frequency and percentage distribution were used to summarize demographic variables. Weighted mean was utilized to assess the level of nurse–nurse collaboration across the four domains of the instrument. Lower mean scores indicated stronger agreement with collaborative behaviors due to reverse scoring of the scale.

Parametric statistical procedures were selected based on the level of measurement and distribution characteristics of the study variables. Normality assessment was conducted through inspection of data distribution characteristics, including assessment of distribution patterns and variability, prior to selection of parametric statistical procedures. Fisher’s exact test was used to examine relationships between gender and collaboration domains, while Pearson correlation analysis was utilized to assess relationships between age, length of clinical experience, and nurse–nurse collaboration. Statistical significance was set at *p* < .05. All submitted questionnaires were reviewed for completeness prior to analysis, and no significant missing data were identified.

### Ethical considerations

Ethical approval was obtained from the Institutional Ethics Review Board of Pamantasan ng Lungsod ng Marikina. Participation in the study was voluntary, and informed consent was obtained from all participants. Confidentiality and anonymity of respondents were strictly maintained throughout the research process.

## Results

### Demographic characteristics

The respondents represented various age groups within the millennial cohort. The largest proportion of respondents were aged 30 years and below (38.7%), followed by respondents aged 34–36 years (15.6%), 37–39 years (15.0%), 31–33 years (13.3%), and 40 years and above (17.3%). Most respondents were female (68.8%) and had 2–9 years of clinical experience (76.9%).

The demographic characteristics of the respondents are presented in (Table 1).

**Table 1:** Demographic characteristics of respondents (*n* = 173)

Variable	Frequency	Percentage
**Age**		
≤ 30 years	67	38.7%
31–33 years	23	13.3%
34–36 years	27	15.6%
37–39 years	26	15.0%
≥ 40 years	30	17.3%
**Sex**		
Female	119	68.8%
Male	54	31.2%
**Length of Experience**		
2–9 years experience	133	76.9%
≥ 10 years experience	40	23.1%

Note: Percentages may not total 100 due to rounding

### Degree of nurse–nurse collaboration

Respondents reported favorable perceptions of nurse–nurse collaboration across all four domains of the Nurse–Nurse Collaboration Scale.

The level of nurse–nurse collaboration across different domains is shown in (Table 2).

**Table 2:** Level of nurse–nurse collaboration among millennial ER nurses

Domain	Mean
Conflict management	1.556
Common goals	1.550
Communication and coordination	1.633
Professionalism and autonomy	1.586

Note: Lower mean scores indicate stronger agreement with collaborative behaviors due to reverse scoring of the instrument. Mean scores closer to 1 indicate more favorable perceptions of nurse–nurse collaboration

### Relationship between demographic variables and collaboration

Pearson correlation analysis showed that length of clinical experience had weak but statistically significant negative relationships with collaboration in terms of common goals and professionalism and autonomy. No significant relationships were observed between collaboration and age or gender.

Fisher’s exact test showed no statistically significant relationship between gender and nurse–nurse collaboration domains (*p* > .05).

The relationship between selected variables and nurse–nurse collaboration is presented in (Table 3).

**Table 3:** Relationship between demographic variables and nurse–nurse collaboration Pearson Correlation Analysis

Variable	Domain	*r*	*p*-value		Interpretation
Length of experience	Common goals	− 0.161		0.034	Significant
Length of experience	Professionalism & autonomy	− 0.178		0.019	Significant
Age	Conflict Management	0.052		0.501	Not significant
Age	Common Goals	− 0.041		0.590	Not significant
Age	Communication and Coordination	0.016		0.833	Not significant
Age	Professionalism and Autonomy	0.077		0.313	Not significant
**Fisher’s Exact Test**					
**Variable**	**Domain**	***r***		***p*** **-value**	**Interpretation**
Gender	Conflict Management	—		> 0.05	Not significant
Gender	Common Goals	—		> 0.05	Not significant
Gender	Communication and Coordination	—		> 0.05	Not significant
Gender	Professionalism and Autonomy	—		> 0.05	Not significant

Note: r = Pearson correlation coefficient; *p* < .05 indicates statistical significance

n = 173 respondents

## Discussion

The findings of this study indicate that millennial emergency room nurses reported favorable perceptions of nurse–nurse collaboration across all measured domains, including conflict management, common goals, communication and coordination, and professionalism and autonomy. These findings highlight the importance of teamwork in emergency healthcare settings where coordinated actions and rapid communication are essential for safe patient care.

Previous studies have similarly reported that effective collaboration among nurses contributes to improved patient outcomes and enhanced workplace satisfaction [5, 20]. Collaborative nursing environments promote open communication, shared problem-solving, and mutual support among healthcare professionals, which are essential for maintaining high standards of patient care in complex clinical environments [21].

The collaborative tendencies observed among millennial emergency room nurses may also reflect characteristics commonly associated with the millennial workforce, including preference for teamwork, open communication, and collaborative learning environments. These generational characteristics may contribute to the favorable perceptions of collaboration observed in the present study.

These findings may support the assumptions of Social Learning Theory, wherein collaborative behaviors are reinforced through repeated workplace interactions, observational learning, and professional role modeling within clinical environments. Emergency room nurses who are continuously exposed to collaborative practices may gradually develop stronger communication patterns and teamwork behaviors through shared clinical experiences.

The absence of significant relationships between collaboration and demographic factors such as age and gender suggests that collaborative behaviors may be influenced more strongly by workplace culture, professional training, and organizational support than by individual characteristics. Similar findings have been reported in previous studies examining teamwork in healthcare settings, where organizational factors such as leadership support, communication systems, and workplace culture were identified as key determinants of collaborative practice [22].

The significant relationship observed between length of clinical experience and certain aspects of collaboration, particularly common goals and professionalism and autonomy, suggests weak negative associations between length of clinical experience and selected domains of nurse–nurse collaboration. These findings may indicate that nurses with longer clinical experience could perceive collaborative interactions differently due to greater professional autonomy, increased workplace responsibilities, or prolonged exposure to stressful emergency care environments [23].

Although statistically significant, the observed correlation coefficients demonstrated weak effect sizes, suggesting that demographic characteristics explained only a limited proportion of variability in collaborative behaviors among respondents. These findings indicate that other organizational and interpersonal factors not directly measured in the study may have stronger influences on nurse–nurse collaboration within emergency healthcare settings.

Emergency departments require strong coordination and communication among healthcare professionals due to the unpredictable nature of patient care demands. Previous research has emphasized that effective teamwork among emergency nurses improves patient safety, enhances clinical efficiency, and reduces medical errors [24]. In such high-pressure environments, collaborative relationships among nurses play a critical role in ensuring timely and coordinated patient care.

In the Philippine healthcare setting, emergency room nurses frequently work in demanding clinical environments characterized by heavy patient loads, workforce shortages, and rapidly changing patient conditions. These workplace realities may intensify the need for effective communication, teamwork, and collaborative decision-making among nurses. Organizational culture, leadership support, and workplace communication practices may therefore play important roles in shaping collaborative behaviors within emergency healthcare teams.

Recent evidence has also highlighted the importance of supportive leadership, resilience-building strategies, and psychologically healthy work environments in strengthening collaborative nursing practice and reducing workplace stress among nurses [8, 25].

Recent studies have also highlighted the influence of workplace environment, management commitment, and safety culture on collaborative nursing behaviors and professional performance within healthcare teams [26, 27].

The findings of this study highlight the importance of fostering collaborative workplace environments through mentorship programs, continuing professional development, and supportive leadership practices. Nursing leaders and healthcare administrators may play a vital role in promoting teamwork by encouraging open communication, conflict resolution skills, and shared decision-making within healthcare teams [28].

### Limitations

This study has several limitations. First, the cross-sectional research design limits the ability to establish causal relationships between demographic characteristics and nurse–nurse collaboration. Second, the study was conducted only among selected government hospitals in Metro Manila, which may limit the generalizability of the findings to other healthcare settings or private institutions. Third, the study relied on self-reported responses, which may introduce response bias, social desirability bias, and common method bias. The uniformly high collaboration scores across domains may also suggest the possibility of ceiling effects or favorable response tendencies among participants. Additionally, organizational factors such as staffing levels, workload, leadership style, and workplace culture were not directly measured but may influence collaborative behaviors among nurses. Future research may consider longitudinal designs, inclusion of organizational variables, and broader healthcare settings to further explore nurse–nurse collaboration.

## Conclusion

Millennial emergency room nurses in selected government hospitals in Metro Manila demonstrated favorable perceptions of nurse–nurse collaboration across all domains. While demographic factors such as age and gender were not significantly associated with collaboration, clinical experience showed weak negative relationships with selected domains of nurse–nurse collaboration. The findings may provide useful insights for nursing leaders and healthcare administrators seeking to support collaborative workplace environments and professional development initiatives within emergency nursing practice.

Strengthening collaborative workplace cultures through supportive leadership, communication training, and professional development initiatives may contribute to safer and more effective emergency nursing practice.

## Data Availability

The datasets used and/or analyzed during the current study are available from the corresponding author on reasonable request.

## Abbreviation

ER: Emergency Room
